# Expressions of actor power in implementation: a qualitative case study of a health service intervention in South Africa

**DOI:** 10.1186/s12913-022-07589-z

**Published:** 2022-02-15

**Authors:** Helen Schneider, Fidele Mukinda, Hanani Tabana, Asha George

**Affiliations:** 1 School of Public Health/SAMRC Health Services To Systems Research Unit, University of the Western Cape, Cape Town, South Africa; 2 School of Public Health, University of the Western Cape, Cape Town, South Africa

**Keywords:** Power, Implementation, Health service intervention

## Abstract

**Background:**

Implementation frameworks and theories acknowledge the role of power as a factor in the adoption (or not) of interventions in health services. Despite this recognition, there is a paucity of evidence on how interventions at the front line of health systems confront or shape existing power relations. This paper reports on a study of actor power in the implementation of an intervention to improve maternal, neonatal and child health care quality and outcomes in a rural district of South Africa.

**Methods:**

A retrospective qualitative case study based on interviews with 34 actors in three ‘implementation units’ – a district hospital and surrounding primary health care services – of the district, selected as purposefully representing full, moderate and low implementation of the intervention, some three years after it was first introduced. Data are analysed using Veneklasen and Miller’s typology of the forms of power – namely ‘power over’, ‘power to’, ‘power within’ and ‘power with’.

**Results:**

Multiple expressions of actor power were evident during implementation and played a plausible role in shaping variable implementation, while the intervention itself acted to change power relations. As expected, a degree of buy-in of managers (with power over) in implementation units was necessary for the intervention to proceed. Beyond this, the ability to mobilise collective action (power with), combined with support from champions with agency (power within) were key to successful implementation. However, local empowerment may pose a threat to hierarchical power (power over) at higher levels (district and provincial) of the system, potentially affecting sustainability.

**Conclusions:**

A systematic approach to the analysis of power in implementation research may provide insights into the fate of interventions. Intervention designs need to consider how they shape power relations, especially where interventions seek to widen participation and responsiveness in local health systems.

## Background

Theories of implementation, whether from the field of policy or implementation science acknowledge the central roles of actors, as individuals and groups, in the adoption of health service interventions. In May’s Normalization Process Theory [1], implementation is a *“process, in which agents intend to bring into operation new or modified practices that are institutionally sanctioned, and are performed by themselves and other agents.”* Similarly, the ﻿Promoting Action on Research Implementation in Health Services (i-PARIHS) framework emphasizes *“groups or teams of individuals [as having] an important role in determining the uptake of new knowledge in practice.”* [2]. Actors play different roles in implementation – amongst others, they can be managers and champions as drivers, brokers and boundary spanners as enablers, external facilitators as catalysts, or frontline providers as targets of intervention [3, 4].

A key attribute of actors is that they have agency, defined as the capacity to *“influence … others with predictable or unpredictable consequences for implementation”* [3]. Agency stems from the interaction of actor values, interests and power and is revealed in how actors adopt, adapt or resist new organisational strategies, even if these are handed down as formal decisions in hierarchies [4]. Agency can be expressed at all levels of the system – from leaders of change to ‘street level bureaucrats’ who are not in positions of authority but who exercise considerable ‘discretionary power’ [5].

This paper explores the phenomenon of actor power in implementation. The exercise of power in health systems is often taken to mean the dynamics of coercion and resistance [6], such as between managers and workers, and the governance mechanisms that address the assymetries of power [7]. However, power can also be understood as a positive force, as proposed by Veneklasen and Miller [8] who define power as *“an individual, collective, and political force that can either undermine or empower citizens and their organizations. It is a force that alternatively can facilitate, hasten, or halt the process of change… its expressions and forms can range from domination and resistance to collaboration and transformation.”* In such a differentiated approach, power emanates from a variety of sources. Apart from overt political, financial (economic) and bureaucratic power, it also resides in professional status and gender norms, and in the knowledge power associated with technical expertise and research, able to shape preferences and discourses [9]. These sources are, in turn, expressed in different *forms* of power, characterised by power *over* (political, economic, hierarchical etc. authority), power *to (“the unique potential of every person to shape his or her life and world”*[8] through factors such as knowledge, skills, experience), power *within (“a person’s sense of self-worth and self-knowledge”* [8], individual agency and psychological capacity to resist internalisation of discrimination) and, finally, power *with (“finding common ground among different interests and building collective strength”* [8] able to engage or challenge other forms of power). The ability of actors to function collectively, in particular, is considered vital to implementation: adopting and assimilating new guidelines, processes or systems requires cooperation and collaboration in the local social orders of the health team, the facility or the district [1, 10, 11].

In their review on the role of power in health systems, Sriram et al. [12] point out that *“understanding and activating power is …. critical to strengthening health systems and improving health outcomes”,* but also understudied. Despite the growing recognition of power as a construct in implementation frameworks [2], research seldom documents how organisational interventions in the front line of health systems shape or confront existing power relations. The issue may be how to ‘see’ power, an ever present but latent phenomenon that is discernable in texts and discourses and the relationships between actors [13], but which becomes more visible during decision-making processes [14] or when seeking to change local practices. Implementation research thus offers a unique opportunity to observe and document the role of power in health systems.

This paper responds to the call for more analyses of power [12, 15], specifically examining its role as a factor in implementation of a health service intervention in South Africa. The intervention concerns an initiative to strengthen accountability for and responsiveness to maternal, neonatal and child deaths in a rural district of South Africa, with the local catchment area of a district hospital and surrounding primary health care clinics and community based services as the basic unit of intervention and analysis. A mixed methods, retrospective evaluation was conducted in the district along with three others targeted by the intervention in 2017, the findings of which are described elsewhere [11, 16, 17].

For this paper, interview data were purposefully selected for further analysis from three local catchment areas in one of the districts representing the range (low, moderate and high) of commitment to the intervention. We aim to explore the forms of individual and collective power in the three units in order to shed light on power as a factor in variable implementation and, conversely, the ways in which the intervention itself (explicitly or implicitly) shaped power relations. From this analysis, we seek to draw conclusions on how a better understanding of power may not only help to explain variation in adoption, but also support the development of interventions that promote empowerment as a key element of their designs.

## Methods

### Design

A retrospective, qualitative embedded case study of expressions of power during the implementation of a health service intervention in three catchment areas (‘implementing units’) of a rural South African district.

### Setting and intervention

One of five districts in a northern province of the country, the study district contains farming areas, small towns and a significant ‘mineral-energy’ complex of mines and coal-fired power stations. At the time of data collection (2017), the district population was around 750,000, the overwhelming majority of whom relied on public health services. Health services are provided in five sub-districts through a mix of hospital, primary health care and community based services (Table 1).

**Table 1:** District profile at time of evaluation (2017)

Population	~ 750,000
Population density	15.5 people/km2
% dependent on the public sector for health care	92.3%
Sub-districts	5
Public health sector facilities	1 Regional Hospital 7 District Hospitals 64 PHC facilities 14 Ward Based Outreach Teams
Per capita annual PHC expenditure in public health system (2016/17)	R837 (US$58)

The district was targeted, with others, by the national Department of Health because of high under-5 and neonatal mortality levels, considered to be retarding progress towards achievement of the Millennium Development Goals. In late 2013, a skilled facilitator, who had previously steered programme implementation as a senior manager in another province, was appointed to support the district. From 2014 onwards, he visited the district once a month, scaling down to every two months after three years.

Key elements of the facilitator-led intervention were new coordination structures, established in each of seven catchment areas (district hospital and surrounding facilities), referred to as Monitoring and Response Units (MRU); a system of real-time (48 h) death reporting, review and response; outreach support from district clinicians and managers; and distribution of evidence-based guidelines. Participants in the MRU, which met monthly, were line managers (referred to as “drivers”), clinician managers (“experts”) and programme managers and information officers (“navigators”), spanning the district hospital, primary health care and community based services. In this regard, the MRU specifically sought to leverage coordinated action on MNCH within the catchment area, crossing official reporting lines which ran in parallel up to the district level. A key principle of the intervention strategy was that no additional funding or external support was to be sourced and that it would rely entirely on better use of existing resources.

By 2017, fairly steep declines in cause-specific under-five mortality, most notably for severe acute child malnutrition, had been recorded in the routine information system of the district, widely attributed by district actors to the effects of the MRU and associated support from district clinicians. The role of the MRU as an intervention in district governance and accountability and the plausible pathways through which it enabled these improved health outcomes are described elsewhere [11].

### Sampling and data collection

Although the MRU was a deliberate system strengthening intervention, it was never set up with research or evaluation in mind. Anecdotal evidence prompted interest from an independent research team (the co-authors), who conducted a post-hoc evaluation three years after the start of implementation. In late 2016, the researchers began observing MRU meetings, reviewed available documents and interviewed the intervention facilitator. From the initial data gathered, key intervention stakeholders were identified and an intervention ‘programme theory’ developed, which formed the basis of further data gathering. In April 2017, the co-authors spent a week in the study district conducting a total of 44 interviews with district and sub-district stakeholders, using a narrative approach, seeking to elicit participants’ understanding and experiences of unfolding implementation (interview guide reported in [17]). A sub-set of interviews from three MRU catchment areas (hereafter referred to as ‘implementing units’) forms the basis of the analysis presented in this paper. The three implementing units were purposefully selected by a knowledegable district programme maternal-child health manager as representing the spectrum (rather than average) of MRU functioning (high, moderate and low) at the time of the evaluation, a judgement corroborated in interview data on MRU meeting frequency and participation in the three sites. The subjective approach to selection was adopted as more objective criteria, such as performance data, failed to reveal any clear patterns.

The three district hospitals ranged in size from 80–143 beds, and were in referral relationships with 8–16 primary health care clinics. A total of 34 actors in the three selected implementing units was interviewed (Table 2). Interviews were set up through the hospital Chief Executive Officer (CEO) with the request to approach the key constituents of the MRU, namely senior and mid-level hospital managers (CEO, nursing service manager, medical manager, maternity and paediatric ward managers, dietitians), primary health care managers, information officers and community outreach team coordinators. The research team worked in pairs, and spent at least one full day in each hospital conducting interviews. Interviews were guided by the programme model, and elements probed included, amongst others, understanding, buy-in to and perceived functioning of the MRU meetings and processes. All interviews were conducted following informed and signed consent, and participation was voluntary. The original study protocol was approved by the University of the Western Cape’s Biomedical Research Ethics Committee and the Provincial Research Committee.

**Table 2:** Actors interviewed in three implementing units

Level	N
Hospital managers (senior and middle)	20
Primary health care managers	8
Community-based teams	4
Other: emergency services, social worker	2
Total	34

### Analysis of data

The original analysis of the full dataset followed the case study approach [18], namely, each unit was first analysed separately and then combined with the others in the district, which was then compared with other districts. A detailed description of the original analysis is described elsewhere [11]. Subsequent, secondary analyses have explored specific mechanisms of change, drawing on theories of enabling environments [17] and governance [11]. This paper is the last in this series, specifically focusing on actor power.

For the power analysis, interviews from the three implementing units were re-analysed, first by listening to the audio recordings (noting the emotional tone of the interview), followed by immersive re-reading of transcripts, then further coding of data into forms of power. ‘Power over’ was taken as the exercise of formal hierarchical authority in the implementation process; ‘power to’ as perceived knowledge and skills in completing work tasks; ‘power within’ as individualised expressions of autonomy or agency, namely *“the ability to make things happen through their own actions”* [1]; and power ‘with’ as evidence of collective action (joint meetings across spheres, subjective reports coordinated action – formal and informal; linked or not to MRU). Manifestations of support for the MRU amongst senior, middle and frontline managers (as representing different levels of authoritative power) were also mapped in a stakeholder analysis [19] of each implementing unit. The three units are referred to in the analysis as ‘full’, ‘moderate’ and ‘low’ implementing units, respectively. As the subject matter could be considered politically sensitive, the names of district and catchment areas are deliberately withheld and identifying data kept to a minimum. In the four years since the evaluation was done, there has been turnover of staff in the three catchment sites and the likelihood of quotes being linked to individuals are minimal.

## Results

Table 3 presents the thematically organised qualitative data, further summarised in the narrative below.

**Table 3:** Expressions of power in high, moderate and low MRU implementing units

Factor	Full implementing unit	Moderate implementing unit	Low implementing unit
*Variable implementation*			
Interviews conducted	14 (plus a group meeting)	13	7
Collective buy-in	- “…one would not hesitate to say that this was one of the best initiatives” (CEO) - “…We cannot do without it. Because it is so important. If it comes to this issue of maternal death, stillbirth rate, the MRU has helped us a lot. Yes. So we really appreciate this MRU programme and we are taking it with two hands.” (maternity OM) -”… this program, I love it…” (paediatric OM) - “…hundred percent I can recommend it.” (dietitian)	-”…I think the MRU is keeping us on our toes.” (NSM) - “I like the MRU, I enjoy it and I think it is yielding results.” (PHC LAM 1) "… when I weigh the pros and cons, we better go with it…it's one helluva job, but believe me, it is worth it." (PHC LAM 2) - “He has planted something… social worker they are responding, dietitian they are responding, nursing side they are responding and clinical manager responding.” (information officer)	- “… there are months where we have skipped [MRU meetings]… and participation is.. somehow it’s not that great” (clinical manager) - “Honestly speaking there isn't a lot of buy-in. There is a confusion between the MRU and [other mortality meetings] … so it was like a duplication of activities… it's taking us away from the focal point of patient care.” (maternity OM) " I don’t know, but I don't think it will be sustainable"(paediatric OM)
Formal authority (power over)	Stable senior and middle managers, active drivers of the MRU “… so I think we owe a lot to our CEO in a sense that he buying in to the idea and he is supporting us.” (clinical manager) “we do have a distributive leadership” (CEO)	High turnover of CEO (3^rd^) and clinical manager, supportive but not active drivers “I heard our CEO—he is still new—I heard him saying I need to attend this MRU because when we say it, it's like "wow it's a nice thing"” (information officer) clinical manager attended when “not busy” (NSM) NSM mostly chaired MRU meetings, with active support from paediatric, maternity and PHC nursing managers	Turnover of CEO, and clinical and middle PHC managers during the period of implementation. Key senior players in ‘acting’ positions, including CEO, and clinical and ward managers. Reportedly disengaged Lack of induction of new staff who “…don’t quite understand the value of MRU or what their role is for that matter.” (clinician) Chairing of MRU meetings was “just a matter of whoever is available” (dietitian)
Self-efficacy (power to)	“it was one of the poor performing hospitals in the entire district. But like now, it’s one of the best.” (paediatric OM) “Those who form part of this I think they are empowered, because now you can see everybody is improving, even in their daily jobs they are active. They know what to do. And they know what to follow, what procedures to do, what policies to implement.” (information officer)	“always it's teaching, it's learning, it's empowering.” (PHC LAM2) “The [existing mortality audit meetings] to me is more like information gathering …as compared to the MRU where you would want to go to the bottom of what happened.” (PHC LAM 1) “I think it is really helping because …now in paeds for pneumonia no death, I don’t know for how long, diarrhoea, no death, there no death from malnutrition I think, ja one, you see it can be one at times it is zero, zero” (information officer)	"if we have one death, we sit down with the doctor, we must find out what is the cause, because we have that [mortality review] programme. Starting from home what happened… [from the] clinic, check road to health booklet, casualty, up to the ward. So we do an improvement plan so we don't repeat" (paediatric OM) District clinical specialists attended the mortality review meetings and were “really helping" (paediatric OM) “our hospital has always encouraged companionship during delivery” (maternity OM)
Agency (power within)	Ability to engage powerful players: “I had constant meetings with the staff at X Clinic, because there were about six mid-wives who resigned, and … the management of the clinic then said no, we are downgrading the hours to 12 h. We then said no, let's engage the district. The district said unfortunately there was a moratorium and I said no, let's write the motivation directly to the office of the MEC [provincial minister of health], and she approved that we can appoint the people” (CEO) Perceived equality: “Don't come and think that you [the CHWs] are subordinate to anybody. You are part of the team, whatever suggestion you have, because you are the people that have a direct contact with the patients, and all the communities” (WBOTs team leader)	Ability to engage powerful players: “Obviously when we are in a situation like this one, pick up the phone whether it is weekend, it’s during the night; call the DCST [district clinical specialist team] if you can.” (NSM) “…I realized this would end up being a maternal death, so I contacted my nurse manager and the nurse manager said “no even contact the CEO”, so I called the CEO, so eventually they accepted the patient and then she was well.” (maternity OM) “…now what I do, after checking [the data], and then I will sit down with them, all of them and then I name and shame [laughs]… now everyone…. the unit managers … they must own their data.” (information officer)	Free to innovate: "I am free to innovate, start projects… many people who have practice the years I have are bored with their practice because they do one and the same thing over and over. So me, I am not bored with my job, because I can always start something new and work on something" (dietitian) Narrative of resistance: “in my view sustaining MRU while we are doing [other mortality audits] is not very beneficial” (maternity OM)
Relationships, team work, collective action (power with)	Inter-professional: “So I think that, that teamwork that is there, that is making everyone come in to want to contribute to the betterment of this community as far as health care is concerned” (clinical manager) Hospital and PHC: “I think it’s the integration between PHC and the hospital. We’ve got an open relationship with each other. You know, if they’ve got a problem at the clinic they will tell you, listen, we’ve got a problem, this is what happened and then it will be fixed” (PHC OM) Collective mindsets: “to be able to have a similar understanding of the primary goal of the whole picture of PHC and the hospital. Because, without it, without the two linked together you wouldn’t be able to achieve what we are able to achieve as far as MRU is concerned.” (clinical manager) Shared resources: “I mobilised my maintenance team and said, for the coming two weeks, you just take the car and go to the clinics, make your own assessment of the facilities. You know, those things that you can do immediately. And those that need replacement like ceilings, then you can just write the motivation – I'll take it to district so the district can assist with the resources” (CEO) “at hospital, not a long while back they were, in need of… surgical… consumables, then at the clinics we will send them to the hospital. Next time we are short of things they will help us…” (PHC OM)	Hospital and PHC: “Immediately they admit a woman they realize that something was not properly done, they pick up the phone quickly and then talk to their partners their colleagues in the clinics so it helps us to be able to care completely for our patients. … it helps us to build strong relationships.” (NSM) "[the hospital] and feeder clinics were not having a relationship, but now, after we engaged with the MRU, we have a relationship which we want to strengthen." (PHC LAM1) Collective mindset: “For some time back we were presenting as PHC as two local areas and the hospital was presenting theirs, however currently we are consolidating our data to be just as one presentation.” (PHC LAM2)	Inter-professional: “We have a group of young health professionals in the hospital, they call themselves the "fresh team", the young ones, it's doctors and allied support staff… they have those open days but they go into communities and address teenagers and try to role model…” (maternity OM) Hospital and PHC: "The dietitians in the hospital and the feeder clinics we have a whattsapp group" (dietitian) PHC “send their subordinates, managers never used to come.” (clinician) “…we discuss confidential issues we wouldn't want WBOTs [ward based outreach teams] to be part of MRU.” (maternity OM) "Last month, we had very decreased admissions in the ward, and they were saying eh-eh our BUR [bed utilisation rate] is going down, as if we are not working… BUR is going down because PHC is doing their work… but if we are closing the tap, the hospital is suffering because we don't admit" (paediatric OM)

*OM* operational manager, *LAM* local area manager, *CEO* chief executive officer, *NSM* nursing service manager, *PHC* primary health care, *WBOT* ward based outreach team

### Collective buy-in and variable implementation

The variable implementation across the three units was confirmed in interviewee accounts of buy-in to MRU, as well as in the ease of doing fieldwork and willingness of respondents (especially senior managers) to engage with the research. In the full implementing unit (IU), the CEO indicated that “one would not hesitate to say that this was one of the best initiatives”. This sentiment was echoed by other key members of the MRU, who also spoke about it in effusive terms: “… we cannot do without it” (maternity manager), “… we love it…” (paediatric ward manager).^1^ The interviewees from the moderate IU, most of whom came from the middle and frontline manager ranks, were more muted in their appraisal, while still expressing support for the initiative (“I think the MRU is keeping us on our toes”), and indicating that MRU meetings were held monthly “without fail”. In contrast, in the low IU, the tone of interviews was mostly one of disengagement, with one respondent openly resisting the MRU. Here the MRU was not perceived to be adding value to established processes of mortality review, and interviewees were of the opinion that the MRU was unlikely to be sustained once the facilitator no longer visited. The research team was able to interview only 7 MRU actors in this IU, and of these, the dietitian was the only one who could be described as enthusiastic. This was possibly because the MRU had specifically enabled a new focus on child malnutrition and had sought to elevate the role of dietitians in the district.

### Exercise of formal authority (power over)

The stability, degree of involvement and support of senior managers was strikingly different in the three units, and clearly impacted on implementation – in the full IU the MRU meetings were chaired by the CEO or the clinical manager, and processes were steered by a combination of senior and middle managers, in a model of leadership described as “distributed” by the CEO. In the moderate IU, there had been leadership turnover, both of CEOs (three in three years) and the clinical manager during the implementation period. The MRU was, however, held together by a critical mass of stable senior and mid-level nursing leadership – the senior nursing services manager (NSM), managers in maternity and paediatric wards, and clinic/primary health care managers (referred to as local area managers—LAMs). The newly appointed CEO was also reported as expressing interest in the MRU initiative. In the low IU, turnover was more extensive, involving senior and middle managers in both the hospital and PHC services, who had been replaced by ‘acting’ managers with reportedly low engagement in the work of the MRU. Chairing of meetings was delegated to lower level managers (“whoever is available”), usually the dietitian or one of the ward managers.

Table 4 summarises the positions (support/neutral/resistant) of senior, middle and frontline managers in the hospital and PHC sevices on the MRU. Frontline (or hybrid) managers are defined as those overseeing and providing clinical care, middle managers as those supporting frontline managers and senior managers as overseeing middle managers. In the full IU, there was buy-in to the MRU across the board and willingness of senior managers to drive the initiative. In the moderate IU, distributed support was also evident but to a lesser degree. In the low IU, there was no obvious engagement with the intervention amongst senior hospital and middle PHC managers, only one proponent in the hospital’s middle management, and one node of active resistance amongst frontline managers.

**Table 4:** Summary of senior, middle and frontline manager positions on the MRU in the three units

	Position on MRU
*Full implementing unit*	
Senior managers	All supportive, and all active drivers
Middle managers	All supportive, one PHC LAM^a^ as key champion
Frontline managers	All supportive, paediatric OM^a^ as key champion
*Moderate implementing unit*	
Senior managers	All (reportedly) supportive, but one main driver (nursing manager)
Middle managers	All supportive, one PHC LAM as champion
Frontline managers	All supportive, maternity OM as champion
*Low implementing unit*	
Senior managers	Senior managers (reportedly) disengaged
Middle managers	One hospital middle manager (senior dietitian) supportive, PHC LAMs (reportedly) disengaged
Frontline managers	One frontline hospital OM supportive, another actively resistant; PHC OM neutral

^a^*LAM* local area manager, *OM* operational manager

### Self-efficacy (power to)

With respect to perceived knowledge and skills in completing work task, interviewees in all three IUs expressed a degree of self-efficacy in their ability to respond to maternal, neonatal and child deaths. The actors in the full IU were described as “empowered” and understood that they had become a “best practice” site. Similarly, in the moderate IU, a sense of self-efficacy was gained from continual processes of learning, and ability to “get to the bottom of problems” and most importantly, reduce mortality. The information officer in this IU – a university graduate – played a key role as the steward of information for the team. In the low IU, structured mortality audits and ongoing support from district clinical specialist teams (along with dietitians) was perceived to have contributed to declining in-hospital mortality from severe acute malnutrition in children. The maternity nursing manager described long standing quality improvement efforts in the hospital including “encouraging companionship” during delivery.^2^

### Expressions of agency (power within)

Expressions of agency or ‘power within’ were most evident in accounts of how actors bypassed the official reporting lines to address problems and successfully navigated hierarchies to meet patient and staff needs. In the moderate IU, for example, senior and mid-level nursing managers described how they would not hesitate to engage the district clinical specialist team or the CEO of the referral facility in cases of an emergency; and the information officer could name and shame a group of unit managers who submitted poor quality data reports. In the full IU, the CEO had managed to secure additional midwife posts for primary health care facilities by appealing directly to the provincial minister of health – jumping over multiple layers of the bureaucracy and challenging hiring freezes. Agency was also reflected in other ways—the freedom to innovate described by the dietitian, the resistance to the MRU expressed by the maternity manager in the low IU and a discourse of equality by a ward based (community) outreach team leader in the full IU. The tone of the interviews – passive, fatalistic vs engaged, motivated – also provided an indirect means to judge agency.

### Relationships, team work, collective action (power with)

Expressions of ‘power with’ in the IUs were related to inter-professional team work (particularly within the hospital setting), but more significantly to the existence of collaborative relationships between hospitals and primary health care services (including community based services), which normally have separate reporting lines to the district. In the full IU these relationships extended beyond open communication and common visions, also present in the moderate IU, to include mutual sharing of material resources. There was a powerful local norm of everyone pulling together. In the low IU, instances of collective action were more fragmented – such as community outreach activities by hospital teams and collaboration between dietitians in the hospital and clinics. At a managerial level there was a disconnect – and even antagonism – between the hospital and PHC services. For example, attendance by community-based staff in mortality review meetings was considered inappropriate; and if PHC services performed well this meant fewer admissions in the paediatric ward and a reduced bed utilisation rate, and the risk of staff being accused of “not working”.

## Discussion

The starting point for this paper was the relevance of actor power as a factor in implementation, in this case a health service intervention in a rural district of South Africa. The analysis adopts the Veneklasen and Miller definition that considers power as both enabling and constraining implementation, and a typology of forms of power that includes hierarchical power (‘power over’), the power of collective action (‘power with’), and the agency of individual actors (‘power within’). Similar to other studies [13], this approach proved useful for identifying and characterising power relations in the context of the intervention. The findings suggest that forms of power (or their absence) and their distribution may explain variation in implementation, while conversely, interventions need to recognise how they engage and shape power relations in diverse ways. As Langley and Denis [20] point out *“however rational and reasonable they may appear on paper, quality improvement initiatives, like other forms of organisational innovation, will fail unless they are designed and implemented in such a way as to take into account the pattern of interests, values and power relationships that surround them.”*

The willingness of those in a hierarchical line authority (power over) to endorse and drive implementation is regarded as a necessary condition for adoption and implementation at other levels [3]. Indeed, the stability and buy-in of the senior managers varied significantly in the three units, with wholesale endorsement by the leadership team in the full IU enabling integration of MRU processes into organisational practice still evident some four years later. The situation in the moderate IU is interesting – here one strong node of support from a stable senior nursing manager, in alliance with middle level and frontline managers in the hospital wards and PHC services, was sufficient to ensure successful implementation. However, in a wider context of high managerial turn over, the reliance on a single driver in the senior management team meant that the intervention was vulnerable in this unit. The MRU failed to gain traction in the low IU where the intervention was perceived as neither necessary nor of added value – a situation of low ‘change valence’ [21].

Beyond formal leadership endorsement, the analysis also highlighted the role of perceived self-efficacy (power to) and agency (power within) as enabling positive action in local health systems. Senior managers who combined line authority with these attributes were able to create significant decision-space [22] around themselves, evident in the ability to mobilise additional resources and advance local collective action in the full IU. The presence of these capacities in middle managers (as brokers) and frontline clinicians (as champions) was manifest in the way they engaged senior managers and in their critiques and narratives of resistance, highlighting the distributed nature of power [23, 24]. Overt expressions of power by lower level players are not without risk, and in the examples identified appeared to reflect not only actor agency (power within) but also a tacit understanding (‘know how’/ ‘know when’) of how to manoevre within organisational hierarchies.

The ways in which the MRU intervention shaped power relations are described in detail elsewhere [11]. Principally, the MRU created a new meso-level governance mechanism that sought to widen participation and accountability in decision-making, across managerial layers, professional boundaries, and levels of the health system. The most significant achievement of the MRU was widely seen as its ability to mobilise collective action (power with) in a local service delivery unit. In the process, the MRU intervention also flattened hierarchies and created new ‘invited spaces’ [25] and mandates for clinicians, middle managers and frontline players to take action on issues related to maternal, neonatal and child health (power to). However, the MRU and other quality interventions premised on collective and responsive decision-making in local health systems could be viewed as a challenge to hierarchical forms of ‘power over’ (especially at higher levels) and as therefore inherently political [20, 26]. While greater participation may lead to widening of support for an intervention amongst middle and frontline players, as in the case of the MRU, these interventions may not survive if power relations at higher levels – in this case district and provincial levels—are not also recast in more enabling ways [27].

### Limitations

A retrospective analysis such as this, derived largely from interviews and seen through the lens of one intervention, is not able to disentangle the cause-effect relationships between power and implementation. It is plausible, as argued, that a priori power relations shaped the variable adoption and implementation of the intervention across the three units, even if the intervention itself shifted relations of power (expressed most concretely in the idea of “empowerment”). However, it is also plausible that other factors were at play. For example, a high turnover of senior managers in two of the units hinted at complex underlying dynamics, whose understanding would have required a more sustained research engagement. In-depth research in a neighbouring province, for example, found that fractious engagements between managers and organised labour were often the source of breakdowns in the ‘negotiated order’ of the local health service [28]. Relationships between the health service and community systems represent a key additional interface of power not addressed in this study [29].

The definitions of the forms of power used in this paper are subject to varying interpretations and there is debate as to whether they are completely distinct or overlapping constructs – such as between ‘power to’ and ‘power within’ [27]. Other studies may operationalise these constructs differently. Similarly, it was not possible to determine how the different forms of power were related to each other and emerged over time, for example, whether power ‘within’ followed power ‘with’ and ‘to’ or vica versa.

Overcoming these various limitations would require prospective designs that carefully track unfolding forms and expressions of power over time.

Finally, it is possible that the unit designations (high, moderate, low implementing) may have introduced selective reading of interviews, a potential analytic bias that inter-subjective agreement within the authorship team sought to minimise.

## Conclusions

This paper has shown how a systematic analysis of power may provide insights into implementation processes, and the need to recognise that health service interventions engage power, especially those interventions that seek to widen participation and responsiveness in local health systems. A differentiated approach to analysing forms of power, as adopted in this study, provides the basis for considering the exercise of power as both a productive and a constraining force. This approach also enables an understanding of power as distributed, exercised by multiple actors in a variety of ways. Interventions that redistribute power (or ‘empower’) in health systems may facilitate ownership but may also be seen as challenging power relations at other levels, and impede sustainability. Implementation actors thus need to recognise the inherently political nature of their work, and the political leadership skills this demands of them.

## Data Availability

This qualitative dataset concerns an intervention associated with specific places and actors, making anonymising and removing any potentially sensitive observations from interview transcripts difficult.

## Abbreviations

CEO: Chief Executive Officer
IU: Implementing unit
LAM: Local Area Manager
MRU: Monitoring and Response Unit
OM: Operational Manager
PHC: Primary Health Care

## Footnotes

1 A visit to the hospital in early 2021, four years after the evaluation revealed that despite some turnover of leadership, the MRU still met regularly and had become a best practice model for other catchment areas. With avoidable deaths an increasingly rare event, the focus was shifting to analysis of ‘near misses’ and avoidable morbidity.

2 In the interview conducted with the district paediatric clinical specialist, he singled out the low IU as in fact a good performer with respect to child mortality.

## References

[1] May C (2013). Towards a general theory of implementation. Implement Sci.

[2] Harvey G, Kitson A (2016). PARIHS revisited: From heuristic to integrated framework for the successful implementation of knowledge into practice. Implement Sci.

[3] Damschroder LJ, Aron DC, Keith RE, Kirsh SR, Alexander JA, Lowery JC (2009). Fostering implementation of health services research findings into practice: A consolidated framework for advancing implementation science. Implement Sci.

[4] Greenhalgh T, Wherton J, Papoutsi C, Lynch J, Hughes G, A’Court C (2017). Beyond adoption: A new framework for theorizing and evaluating nonadoption, abandonment, and challenges to the scale-up, spread, and sustainability of health and care technologies. J Med Internet Res.

[5] Gilson L, Schneider H, Orgill M (2014). Practice and power: A review and interpretive synthesis focused on the exercise of discretionary power in policy implementation by front-line providers and managers. Health Policy Plan.

[6] Moon S (2019). Power in global governance: an expanded typology from global health. Global Health.

[7] The World Bank (2017). Governance and The Law: World Development Report 2017.

[8] VeneKlasen I, Miller V (2007). A New Weave of Power, People & Politics: An Action Guide for Advocacy and Citizen Participation.

[9] Dalglish SL, Surkan PJ, Diarra A, Harouna A, Bennett S (2015). Power and pro-poor policies: The case of iCCM in Niger. Health Policy Plan.

[10] Fligstein N (2001). Social Skill and the Theory of Fields. Sociol Theory.

[11] Schneider H, George A, Mukinda F, Tabana H (2020). District Governance and Improved Maternal, Neonatal and Child Health in South Africa: Pathways of Change. Heal Syst Reform.

[12] Sriram V, Topp SM, Schaaf M, Mishra A, Flores W, Rajasulochana SR (2018). 10 Best Resources on Power in Health Policy and Systems in Low- and Middle-Income Countries. Health Policy Plan.

[13] Lehmann U, Gilson L (2013). Actor interfaces and practices of power in a community health worker programme: A South African study of unintended policy outcomes. Health Policy Plan.

[14] Barasa EW, Cleary S, English M, Molyneux S (2016). The influence of power and actor relations on priority setting and resource allocation practices at the hospital level in Kenya: a case study. BMC Health Serv Res.

[15] Gore R, Parker R (2019). Analysing power and politics in health policies and systems. Glob Public Health.

[16] Schneider H, McKenzie A, Tabana H, Mukinda F, George A (2017). Evaluation of health system strengthening initiatives for improving the quality and outcomes of maternal, neonatal and child health care in four South African districts.

[17] Schneider H, Van Der Merwe M, Marutla B, Cupido J, Kauchali S (2019). The whole is more than the sum of the parts: Establishing an enabling health system environment for reducing acute child malnutrition in a rural South African district. Health Policy Plan.

[18] Yin RK (2014). Case study research: Design and methods.

[19] Varvasovszky Z, Brugha R (2000). How to do (or not to do)...: A stakeholder analysis. Health Policy Plan.

[20] Langley A, Denis JL (2011). Beyond evidence: The micropolitics of improvement. BMJ Qual Saf.

[21] Weiner BJ (2009). A theory of organizational readiness for change. Implement Sci.

[22] Bossert T (1998). Analyzing the decentralization of health systems in developing countries: decision space, innovation and performance. Soc Sci Med.

[23] Parashar R, Gawde N, Gupt A, Gilson L (2020). Unpacking the implementation blackbox using ’actor interface analysis’: How did actor relations and practices of power influence delivery of a free entitlement health policy in India?. Health Policy Plan.

[24] Fitzgerald L, Ferlie E, McGivern G, Buchanan D (2013). Distributed leadership patterns and service improvement: Evidence and argument from English healthcare. Leadersh Q.

[25] Gaventa J (2006). Finding the spaces for change: A power analysis. IDS Bull.

[26] Gilson L (2016). Everyday politics and the leadership of health policy implementation. Heal Syst Reform.

[27] Pansardi P, Bindi M (2021). The new concepts of power? Power-over, power-to and power-with. J Polit Power.

[28] Mukinda FK, Van Belle S, Schneider H (2020). Perceptions and experiences of frontline health managers and providers on accountability in a South African health district. Int J Equity Health.

[29] Gore R (2019). The power of popular opinion in everyday primary care provision in urban India. Glob Public Health.

